# Small angle neutron scattering data of polymer electrolyte membranes partially swollen in water

**DOI:** 10.1016/j.dib.2016.03.011

**Published:** 2016-03-09

**Authors:** Yue Zhao, Miru Yoshida, Tatsuya Oshima, Satoshi Koizumi, Masahiro Rikukawa, Noemi Szekely, Aurel Radulescu, Dieter Richter

**Affiliations:** a Quantum Beam Science Center (QuBS), Japan Atomic Energy Agency (JAEA), Watanuki-machi 1233, Takasaki, Gunma 370-1292, Japan; b Department of Material Science, Sophia University, Tokyo 102-0094, Japan; c Department of Engineering, Ibaraki University, Hitachi 615-8510, Japan; d Forschungszentrum Jülich GmbH, Jülich Centre for Neutron Science @ MLZ, Lichtenbergstraße 1, D-85747 Garching, Germany; e Jülich Centre for Neutron Science & Institute for Complex Systems, Forschungszentrum Jülich GmbH, D-52425 Jülich, Germany

**Keywords:** Small angle neutron scattering, Polymer electrolyte membranes, Hard-sphere model

## Abstract

In this article, we show the small-angle neutron scattering (SANS) data obtained from the polymer electrolyte membranes (PEMs) equilibrated at a given relative humidity. We apply Hard-Sphere (HS) structure model with Percus–Yervick interference interactions to analyze the dataset. The molecular structure of these PEMs and the morphologies of the fully water-swollen membranes have been elucidated by Zhao et al. “Elucidation of the morphology of the hydrocarbon multi-block copolymer electrolyte membranes for proton exchange fuel cells” [1].

**Specifications Table**

**Table:** 

Subject area	*Materials science*
More specific subject area	*Soft matter*
Type of data	*Table, figure*
How data was acquired	*Small angle neutron scattering instrument at KWS2, FRM2*
Data format	*Analyzed*
Experimental factors	The dry membranes with an average thickness of ~50 μm were prepared by solution casting onto a flat glass plate from its dimethyl sulfoxide solution with a concentration of 5 wt%. Partially water swollen membranes were prepared by putting the dry membranes into a humility controller at 30% relative humidity and 25 °C.
Experimental features	The incident neutron beam was monochromatized with a velocity selector to have the average wavelength (*λ*) of 5 Å with a wavelength resolution of Δ*λ/λ*= 20%. All of the measurements were done at 25±0.5 °C. The scattering patterns were collected with a two-dimensional scintillation detector, and circularly averaged to obtain scattering intensity profiles as a function of *q*, where *q* is the scattering vector, defined as *q*=(4*π*/*λ*)sin(*θ*/2) with *θ* being the scattering angle. The scattering profiles were corrected for the instrument background, detector sensitivity, and scattering from empty cell, and finally calibrated on the absolute scale (cm^−1^) using a Plexiglas secondary standard.
Data source location	SANS measurements were performed with KWS-2 at the neutron source Heinz Maier-Leibnitz (FRM II reactor) in Garching, Germany.
Data accessibility	*Data is with this article*

**Value of the data**

• Hard-sphere structure model is introduced to elucidate the morphology of polymer electrolyte membranes.
• Data of partially swollen membranes together with that of fully swollen membranes leads to a thorough understanding of the morphology.
• The method and model analysis are worthy being applied to other types of membranes.

## 1 Data

Partially water swollen membranes were prepared by putting the dry PEMs into a humility controller at 30% relative humidity and 25 °C. The SANS measurements were performed with KWS-2 at the neutron source Heinz Maier-Leibnitz (FRM II reactor) in Garching, Germany, and the scattering intensity profiles has been corrected and calibrated on the absolute scale (cm^−1^).

**Fig. 1:**
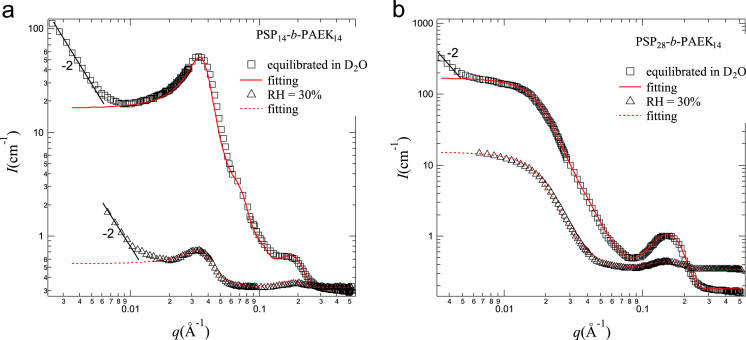
Part (a) SANS profiles of PSP_14_-*b*-PAEK_14_ membranes equilibrated at RH=30% (triangles) and fully D_2_O-swollen state (squares) at room temperature. The best-fitted theoretical curves ranging from the middle-*q* region based on HS model to the high-*q* region based on Eq. (6) for both membranes are also shown in the figure by red dashed and solid lines, respectively. Part (b) SANS profiles of PSP_28_-*b*-PAEK_14_ membranes equilibrated at RH=30% (triangles) and fully D_2_O-swollen state (squares) at room temperature. The best-fitted theoretical curves ranging from the middle-*q* region based on HS model to the high-*q* region based on Eq. (6) for both membranes are also shown in the figure by red dashed and solid lines, respectively.

Fig. 1a and b show the SANS intensity profiles of the two membranes, PSP_14_-*b*-PAEK_14_ and PSP_28_-*b*-PAEK_14_, as a function of scattering vector *q*, respectively. The profile of the corresponding fully D_2_O-swollen membranes is plotted in the same figure as a reference. Hard-Sphere (HS) structure model with Percus–Yervick interference interactions was applied to analyze these scattering profiles [1], [2]. The best fitting parameters are listed in Tables 1 and 2. Note that the profiles at high-*q* range (0.08<*q*<0.45 Å^−1^) can be fitted well by Eq. (6) below, and the best fitted curve is summed up with the fitting curve in the middle-*q* range and shown in the figure.

## 2 Experimental design, materials and methods

### 2.1 Materials

Two multiblock copolymer poly(sulfonate phenylene)-*b*-poly(arylene ether ketone) with different block ratios, designated as PSP_14_-*b*-PAEK_14_ and PSP_28_-*b*-PAEK_14_ for brevity, were synthesized by varying the stoichiometry of the sulfonated monomers and hydrophobic oligomers via the nickel-catalyzed polymerization [3], [4]. The subscript 14 or 28 refers to the repeating unit number in each block. The molecular structure and characteristics of these two polymers can be found elsewhere [1], [2]. The dry membranes with an average thickness of ~ 50 μm were prepared by solution casting onto a flat glass plate from its dimethyl sulfoxide solution with a concentration of 5 wt% [3]. Partially water swollen membranes were prepared by putting the dry membranes into a humility controller at 30% relative humidity and 25 °C.

### 2.2 Methods

SANS measurements were performed with KWS-2 at the neutron source Heinz Maier-Leibnitz (FRM II reactor) in Garching, Germany [5]. The incident neutron beam was monochromatized with a velocity selector to have the average wavelength (*λ*) of 5 Å with a wavelength resolution of Δ*λ/λ*= 20%. All of the measurements were done at 25±0.5 °C. The scattering patterns were collected with a two-dimensional scintillation detector, and circularly averaged to obtain scattering intensity profiles as a function of *q*, where *q* is the scattering vector, defined as *q*=(4 *π*/*λ*)sin(*θ*/2) with *θ* being the scattering angle. The scattering profiles were corrected for the instrument background, detector sensitivity, and scattering from empty cell, and finally calibrated on the absolute scale (cm^−1^) using a Plexiglas secondary standard.

### 2.3 Analysis

We assume that the topology of the swollen membranes can be described by an almost random distribution of *n* particles in a homogeneous matrix. Let Δ*b* be the contrast of the particle density with respect to the matrix density and *v* be the of average volume of a single particle, then the observed scattering intensity, *I*(*q*), is [6]

(1) $$I(q)={\left(\Delta b\right)}^{2}nv^{2}P(q)S(q)=KP(q)S(q)$$

where *P*(*q*) is the form factor of the particles, *S*(*q*) is an approximate interference factor and *K* is a constant in terms of Δ*b*, *n* and *v*. We assume that the number of the particles per volume is high that *S*(*q*) must be considered despite the random arrangement of the particles. The contrast Δ*b=b*_*p*_*−b*_*m*_ is defined by the difference between the scattering length density (SLD) of the particle phase, *b_p_*, and that of the matrix phase, *b_m_*. Thus, Δ*b* is computable as long as the shape and composition of the particle phase and the matrix phase are well determined, and their SLDs are theoretically estimated below.

SLD of a molecule of *i* atoms is related to its molecular structure and may be readily calculated from the simple expression given by $b=\sum_{i}b_{i}\frac{dN_{A}}{M_{w}}$ where *b*_*i*_ is the scattering length of *i*th atom, *d* is the mass density of the scattering body, *M*_*w*_ is the molecular weight, and *N_A_* is the Avogadoro constant [6].

Let us consider an ensemble of spheres with varying sizes that can be described by a Gaussian size distribution:

(2) $$P\left(q\right)=\int_{0}^{\infty }{\left\{\frac{3}{{\left(qr\right)}^{3}}\left[\sin\left(qr\right)-qr\;cos\left(qr\right)\right]\right\}}^{2}\frac{1}{{\left(2\pi \right)}^{1/2}\sigma_{R}}\exp[\frac{-{\left(r-R\right)}^{2}}{2{\sigma_{R}}^{2}}]dr$$

with *R* being the average radius, and *σ*_*R*_ being its standard deviation. Thus *v*=$\frac{4\pi R^{3}}{3}$. We consider Percus–Yevick expression to account for interparticle interference [2], [7], then *S*(*q*) is the interference factor, described for a random arrangement of spheres by the following expression:

(3) $$S\left(q,R,\phi \;\right)=\frac{1}{1+24\phi (\frac{F\left(A\right)}{A})}$$

here *A*=2*qR* and *ϕ* is the hard sphere volume fraction. *F*(*A*) is a trigonometric function of *A* and *ϕ* given by

(4) $$F\left(A\right)=\frac{\alpha }{A^{2}}\left(sinA-Acos\;A\right)+\frac{\beta }{A^{3}}\left(2A\;sin\;A+\left(2-A^{2}\right)cos\;A-2\right)+\frac{\gamma }{A^{5}}\left(-A^{4}\;cos\;A+4[\left(3A^{2}-6\right)cos\;A+\left(A^{3}-6A\right)sin\;A+6]\right)$$

(5) $$\begin{array}{l} \alpha ={\left(1+2\phi \right)}^{2}\text{/}{\left(1-\phi \right)}^{4}\\ \beta =-6\phi {\left(1+\frac{\phi }{2}\right)}^{2}\text{/}(1-{\phi )}^{4}\\ \gamma =\frac{1}{2\phi }{\left(1+2\phi \right)}^{2}\text{/}(1-{\phi )}^{4} \end{array}$$

The distribution of the ionic clusters at high-*q* range can be fitted well by Gaussian distribution function, where the scattering intensity around the ionomer peak at 0.08 Å^−1^<*q*<0.45 Å^−1^, *I*_ion_(*q*), can be expressed by

(6) $$I_{ion}\left(q\right)=I_{m,ion}G\left(q\right)+\;I_{inc}$$

where *I*_*m*,*ion*_ is the ionomer peak height, *G*(*q*) is Gaussian distribution function about the ionomer peak at *q*_*m*,*ion*_, given by $G\left(q\right)=\frac{1}{{\left(2\pi \right)}^{1/2}\sigma_{q}}\exp[-{\left(q-q_{m,ion}\right)}^{2}/(2{\sigma_{q}}^{2})]$, with *σ*_*q*_ being the standard deviation of *q*_*m*,*ion*_, and *I_inc_* is the incoherent scattering intensity, which can be determined by the average intensity of the flat part of the profile at *q*>0.4 Å^−1^ in the high-*q* region. Eq. (6) is used to fit profiles in Fig. 1a and b and the fitting parameters are listed in Tables 1 and 2.

## Figures and Tables

**Table 2:** Parameters used to fit SANS data of PSP_28_-*b*-PAEK_14_ membranes equilibrated at RH=30% and in D_2_O by Eqs. (1), (6).

PSP_14_-*b*-PAEK_14_	Middle-*q* range (HS model)				High-*q* range (ionomer peak)		
*ϕ*	*R* (Å)	*σ*_*R*_/*R*	*K*	*I*_m,ion_	*q*_m,ion_ (Å^−1^)	*σ*_*q*_/*q*_m,ion_
Equilibrated at RH=30%	0.08	150	0.243	25.9	0.01	0.152	0.243
Equilibrated in D_2_O	0.07	145	0.245	295.3	0.08	0.152	0.243

**Table 1:** Parameters used to fit SANS data of PSP_14_-*b*-PAEK_14_ membranes equilibrated at RH=30% and in D_2_O by Eqs. (1), (6).

PSP_14_-*b*-PAEK_14_	Middle-*q* range (HS model)				High-*q* range (ionomer peak)		
*ϕ*	*R* (Å)	*σ*_*R*_/*R*	*K*	*I*_m,ion_	*q*_m,ion_ (Å^−1^)	*σ*_*q*_/*q*_m,ion_
Equilibrated at RH=30%	0.25	80	0.247	1.56	0.004	0.18	0.194
Equilibrated in D_2_O	0.32	85	0.247	211.3	0.026	0.18	0.194

## References

[1] Zhao Y., Yoshida M., Oshima T., Koizumi S., Rikukawa M., Szekely N., Radulescu A., Richter D. (2016). Elucidation of the morphology of the hydrocarbon multi-block copolymer electrolyte membranes for proton exchange fuel cells. Polymer.

[2] Percus J.K., Yevich G.J. (1958). Analysis of classical statistical mechanics by means of collective coordinates. Phys. Rev..

[3] Yoshida M., Zhao Y., Yoshizawa-Fujita M., Ohira A., Takeoka Y., Koizumi S., Rikukawa M. (2013). PFG-NMR and SANS studies in cation exchange membranes based on sulfonated polyphenylene multiblock copolymers. ECS Trans..

[4] Tonozuka I., Yoshida M., Kaneko K., Takeoka Y., Rikukawa M. (2011). Considerations of polymerization method and molecular weight for proton-conducting poly(p-phenylene) derivatives. Polymer.

[5] Radulescu A., Pipich V., Frielinghaus H., Appavou M.S. (2012). KWS-2, the high intensity/wide Q-range small-angle neutron diffractometer for soft-matter and biology at FRM II. J. Phys.: Conf. Ser..

[6] Roe R.J. (2000). Methods of X-ray abd Neutron Scattering in Polymer Science.

[7] Kinning D.J., Thomas E.L. (1984). Hard-sphere interactions between spherical domains in diblock copolymers. Macromolecules.

