# Supplementary material for: Small angle neutron scattering data of polymer electrolyte membranes partially swollen in water
Source: Data Brief. 2016 Mar 9;7:599–603. doi: 10.1016/j.dib.2016.03.011 (PMC4802542; doi:10.1016/j.dib.2016.03.011)
Supplement: Supplementary material [file mmc1.doc]

*Quantum Beam Science Directorate, Japan Atomic Energy Agency*

*1233 Watanuki, Takasaki, Gunma 370-1292, Japan*

***Yue Zhao***

**Tel**: +81-27-346-9100 **Fax**: +81-27-346-9687

**E-mail**: [zhao.yue@jaea.go.jp](mailto:zhao.yue@jaea.go.jp)

Feb. 15, 2016

Conflicts of interest: none

Yue Zhao
